# Aberrant mitochondrial homeostasis at the crossroad of musculoskeletal ageing and non-small cell lung cancer

**DOI:** 10.1371/journal.pone.0273766

**Published:** 2022-09-06

**Authors:** Konstantinos Prokopidis, Panagiotis Giannos, Oliver C. Witard, Daniel Peckham, Theocharis Ispoglou

**Affiliations:** 1 Society of Meta-Research and Biomedical Innovation, London, United Kingdom; 2 Department of Musculoskeletal Biology, Institute of Life Course and Medical Sciences, University of Liverpool, Liverpool, United Kingdom; 3 Department of Life Sciences, Faculty of Natural Sciences, Imperial College London, London, United Kingdom; 4 Faculty of Life Sciences and Medicine, Centre for Human and Applied Physiological Sciences, King’s College London, London, United Kingdom; 5 Leeds Institute of Medical Research at St James’s, University of Leeds, Leeds, United Kingdom; 6 Carnegie School of Sport, Leeds Beckett University, Leeds, United Kingdom; University of South Alabama, UNITED STATES

## Abstract

Cancer cachexia is accompanied by muscle atrophy, sharing multiple common catabolic pathways with sarcopenia, including mitochondrial dysfunction. This study investigated gene expression from skeletal muscle tissues of older healthy adults, who are at risk of age-related sarcopenia, to identify potential gene biomarkers whose dysregulated expression and protein interference were involved in non-small cell lung cancer (NSCLC). Screening of the literature resulted in 14 microarray datasets (GSE25941, GSE28392, GSE28422, GSE47881, GSE47969, GSE59880 in musculoskeletal ageing; GSE118370, GSE33532, GSE19804, GSE18842, GSE27262, GSE19188, GSE31210, GSE40791 in NSCLC). Differentially expressed genes (DEGs) were used to construct protein-protein interaction networks and retrieve clustering gene modules. Overlapping module DEGs were ranked based on 11 topological algorithms and were correlated with prognosis, tissue expression, and tumour purity in NSCLC. The analysis revealed that the dysregulated expression of the mammalian mitochondrial ribosomal proteins, Mitochondrial Ribosomal Protein S26 (MRPS26), Mitochondrial Ribosomal Protein S17 (MRPS17), Mitochondrial Ribosomal Protein L18 (MRPL18) and Mitochondrial Ribosomal Protein L51 (MRPL51) were linked to reduced survival and tumour purity in NSCLC while tissue expression of the same genes followed an opposite direction in healthy older adults. These results support a potential link between the mitochondrial ribosomal microenvironment in ageing muscle and NSCLC. Further studies comparing changes in sarcopenia and NSCLC associated cachexia are warranted.

## Introduction

Sarcopenia is a muscle wasting disorder characterised by a decline in skeletal muscle mass, strength, and physical function [1]. Rates of sarcopenia are exacerbated with ageing [2] and are prevalent in both healthy community dwelling older adults as well as those in long-term care. The estimated prevalence of sarcopenia varies significantly but can be as high as ~40% in healthy older adults [3, 4] and up to ~75% in older patients [5–7] depending on the diagnostic criteria used. Although the development of sarcopenia is a common phenomenon in ageing, the catabolic responses underpinning skeletal muscle dysfunction have been associated with multiple malignant co-morbidities, including cancer [8].

Similarly to sarcopenia, cancer cachexia is characterised by muscle loss, which can occur with or without the presence of fat loss and is typically associated with symptoms of cachexia, anorexia, fatigue, and early satiety [9]. Common mechanisms shared by both sarcopenia and cancer cachexia, include systemic low-grade inflammation, mitochondrial dysfunction, dysregulated autophagy, cellular senescence, impaired muscle cell regeneration, and higher protein turnover leading to anabolic resistance [10]. In older cancer populations, similar rates of cancer cachexia (~65%) and sarcopenia (as high as ~60%) have been reported [11]. These physiological perturbations may lead to muscle atrophy, compromised innate immunity, increasing physical malfunction, and reducing quality of life [9, 12].

Worldwide, lung cancer is the most prevalent cancer and the leading cause of cancer-related deaths [13]. The pooled prevalence of sarcopenia in non-small cell lung cancer (NSCLC) patients is 52% and ranks first among all cancers and is linked to poor clinical outcomes and survival rates [14]. Interestingly, muscle mass constitutes a prognostic factor during palliative chemotherapy treatment in patients with advanced NSCLC [15]. At present, exercise and nutrition interventions have been widely used for the treatment of NSCLC-related sarcopenia with the aim of improving outcomes [16]. The synergistic effects of exercise and optimisation of protein and energy intake is reported as being paramount, but not exclusive, for the successful management of sarcopenia [17]. With regards to cachexia, adequate nutritional support appears to be the main means of treatment [9]. Exercise [18] and nutrition (i.e., essential amino acids) [19] interventions can be effective complementary non-pharmacological therapies in cancer by preventing and better managing mitochondrial dysfunction [20], an important contributing factor to sarcopenia [21]. However, evidence is limited, and further research is needed to identify successful interventional treatments and markers of sarcopenia progression in association with ageing and NSCLC. The development of targeted interventions requires a greater understanding of the relationship between the pathophysiological mechanisms driving ageing associated sarcopenia and NSCLC cachexia. One important avenue is to identify genetic markers that act as mediators in both conditions [22].

NSCLC cachexia is characterized by gene regulatory alterations underlying muscle wasting [23, 24]. To date, there is a scarcity of published research comparing gene expression in skeletal muscle from healthy older adults *vs* younger adults, and in NSCLC lung tissue *vs* age-matched controls, which could shed light on potential molecular mechanisms driving cachexia and musculoskeletal ageing. Mitochondrial bioenergetic dysfunction is a strong molecular signature of sarcopenia [21], and animal studies, have demonstrated a link between ageing and an increase in the expression of genes involved in inflammation, a key characteristic of both sarcopenia and cancer cachexia [25]. Equally, mitochondrial oxidative phosphorylation (OXPHOS) capacity is reduced in cancer cells and reliance on glycolysis is further increased [26]. Furthermore, studies have revealed localized polymorphisms at nucleotides of mitochondrial tRNA genes [27] and mitochondrial DNA mutations in lung cancer [28, 29] which may be implicated with metastasis-specific lethality [30, 31]. These findings, highlight the importance of identifying how ageing influences gene expression, in older adults, without or with diseases such as cancer, where sarcopenia/cachexia rates [11] are highly prevalent and inflammation is a critical component of tumour progression [32]. Our study focused on examining gene expression from lung tissues of patients with NSCLC and skeletal muscle tissues of older healthy adults. The aim of this study was to identify potential gene markers whose dysregulated expression and protein interaction interference were involved in NSCLC cachexia and musculoskeletal ageing.

## Methods

**Fig 1:**
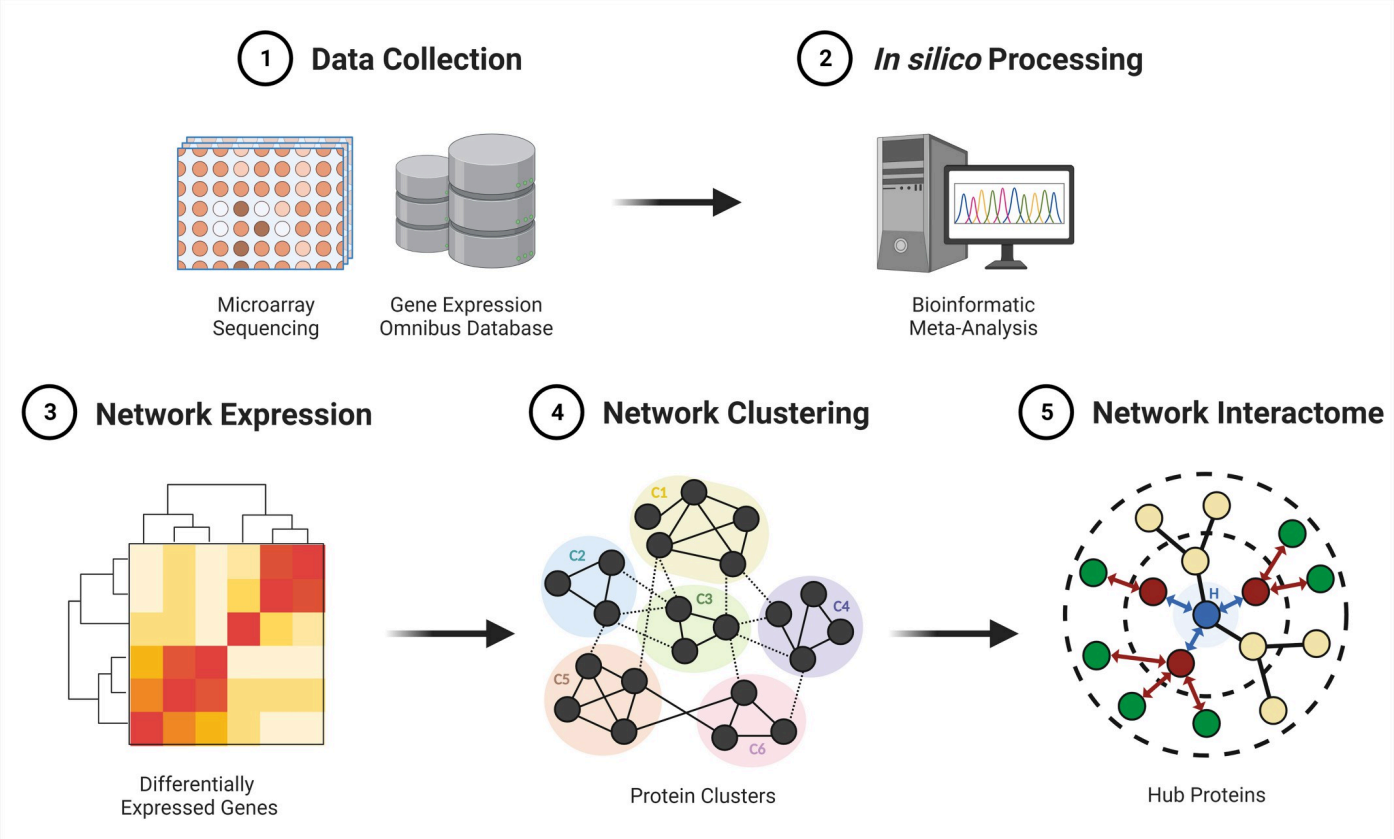
Methodological summary of the stepwise workflow employed in our study. The literature was initially screened through the Gene Expression Omnibus database for publicly available microarray datasets containing skeletal muscle samples from older and lung samples from patients with non-small cell lung cancer (NSCLC) (1). Eligible gene expression profiles were thereafter integrated and meta-analysed using the random effect model (2). Thenceforth differentially expressed genes (DEGs) with the strongest average effect across all included datasets were derived (3). Highly clustered (C) proteins of significant and overlapping DEGs between the two conditions were identified using the Molecular Complex Detection and mapped using The Search Tool for the Retrieval of Interacting Genes (4). The interactome interference of the shared sub-networks was evaluated using the intersection of 11 local and global-based topological algorithms from CytoHubba and central hub objects as potential markers of musculoskeletal ageing and NSCLC disease progression were derived (5). C: Cluster; H: Hub.

A methodological stepwise approach was employed to address the objective of our study (Fig 1).

### Collection of microarray datasets

Screening of the literature was ensued from inception until November 2021, by searching the National Center for Biotechnology Information Gene Expression Omnibus using the search terms: ageing OR aging OR old* OR sarcopenia AND skeletal muscle OR musculoskeletal AND non-small cell lung cancer OR NSCLC OR lung adenocarcinoma OR LUAD OR lung squamous cell carcinoma OR LUSC. An additional search was conducted using the National Library of Medicine PubMed via the following extra terms: differentially expressed genes OR DEGs.

Datasets were restricted based on organism type (Homo sapiens), expression profiling (microarray), sample type (skeletal muscle or lung tissue) and condition (ageing and NSCLC). No restrictions in terms of language and geographic region were applied and datasets without expression data for controls were excluded. No further exclusion criteria pertained to the baseline characteristics of participants from which tissue sections were retrieved, were used.

### Identification of differentially expressed genes

Skeletal muscle samples from older adults (aged ≥ 60 years) were compared to healthy young subjects (aged ≤ 30 years), while lung samples from patients with NSCLC were compared to those from healthy controls who were either age-matched or matched adjacent/distant normal lung tissue from the same patient. No age restrictions were applied to the NSCLC cohort. Differentially expressed genes (DEGs) were retrieved using ImaGEO and the random effect model was employed in the integration of differential gene expression [33]. Genes with the strongest average effect among all included datasets were identified. DEGs following *P*<0.05 corrected by the Benjamini-Hochberg False Discovery Rate were retrieved as significant and those with *Z* score>1.96 were classified as upregulated, while those with *Z* score<-1.96 as downregulated (both corresponding to a 5% significance level).

### Construction of protein-protein interaction networks

DEGs from musculoskeletal ageing and NSCLC were used to create two distinct networks of encoded proteins using The Search Tool for the Retrieval of Interacting Genes (STRING) [34]. The protein-protein interactions (PPI) in the networks were predicted using a medium probabilistic confidence score of >0.4 and constructed with Cytoscape [35]. Applying a reasonably moderate cut-off threshold was employed to enhance the coverage of all potential protein interactions but without overestimating their precision. Proteins lacking interactions were excluded from the networks.

### Identification of clustering modules and hub genes

Highly clustered genes or modules in the PPI networks were retrieved using the Molecular Complex Detection (MCODE) [36]. Application of cut-off was ensued after manual inspection of clusters and a score yielding distinct separation of clusters into groups, was regarded. Clusters with MCODE score >15 were considered as significant modules.

The interactome of module DEGs in the PPI networks was evaluated using CytoHubba based on the intersection of 11 local and global-based topological algorithms as established by Chin *et al*. [37], namely: Degree, Closeness, Betweenness, Radiality, Stress, EcCentricity, BottleNeck, Edge Percolated Component, Maximum Neighborhood Component, Density of Maximum Neighborhood Component and Maximal Clique Centrality. The five highest-ranked module DEGs that overlapped in the musculoskeletal ageing and NSCLC networks, were regarded as hub genes.

### Analysis of prognosis, expression level and tumour purity of musculoskeletal ageing hub genes in NSCLC

The prognostic significance of hub genes common to musculoskeletal ageing and NSCLC, in terms of expression and interactions, were examined in publicly available NSCLC transcriptome data from GEO (GSE14814, GSE19188, GSE29013, GSE30219, GSE31210, GSE3141, GSE31908, GSE37745, GSE43580, GSE4573, GSE50081, GSE8894), TCGA and caArray databases using the Kaplan-Meier-plotter [38]. Patients with NSCLC (*n* = 1927) were divided into high and low expression groups and correlation with overall survival (OS) was retrieved using a log-rank *P*<0.05. Their differential expression in NSCLC was determined using TCGA data via the Gene Expression Profiling Interactive Analysis 2 [39] and the Tumour Immune Estimation Resource 2 [40] algorithms. Overall expression, across different stages and in terms of tumour microenvironment purity in NSCLC tissues was determined based on analysis of variance, a Wilcoxon test *P*<0.05 and the partial Spearman’s correlation (partial rho). Hub genes with significantly reduced OS and altered (but opposite to musculoskeletal ageing) expression in NSCLC, were considered significant and presented as potential gene markers of NSCLC progression in musculoskeletal ageing.

## Results

### Overview of microarray datasets

Our literature search of the GEO and PubMed databases resulted in 14 microarray datasets [GSE25941 [41], GSE28392 [41], GSE28422 [41], GSE47881 [41, 42], GSE47969 [42, 43], GSE59880 [43–45] in musculoskeletal ageing; GSE118370 [46], GSE33532 [47], GSE19804 [48, 49], GSE18842 [50], GSE27262 [51, 52], GSE19188 [53], GSE31210 [54, 55], GSE40791 [56] in NSCLC] (S1 Table). The former datasets included skeletal (*vastus lateralis*) muscle tissue biopsies from healthy young subjects (*n* = 96) and older adults (*n* = 110). The latter datasets included lung tissue [majorly lung adenocarcinoma (LUAD) and lung squamous cell carcinoma (LUSC)] biopsies from healthy controls (*n* = 341) and patients with NSCLC (*n* = 628).

### Differentially expressed genes in musculoskeletal ageing and NSCLC

A total of 1960 DEGs were identified in older individuals when compared to younger counterparts (S2 Table). Of these, 1262 DEGs were upregulated, and 698 were downregulated. In contrast, a total of 4387 DEGs were retrieved in patients with NSCLC when compared to healthy controls, of which 2654 were upregulated and 1733 were downregulated (S3 Table). Comparative analysis between these expression profiles, revealed 540 (9.3%) overlapping DEGs, 1420 (24.5%) unique to musculoskeletal ageing samples and 3847 (66.2%) to NSCLC ones (S4 Table).

### Protein-protein interaction networks and modules in musculoskeletal ageing and NSCLC

**Fig 2:**
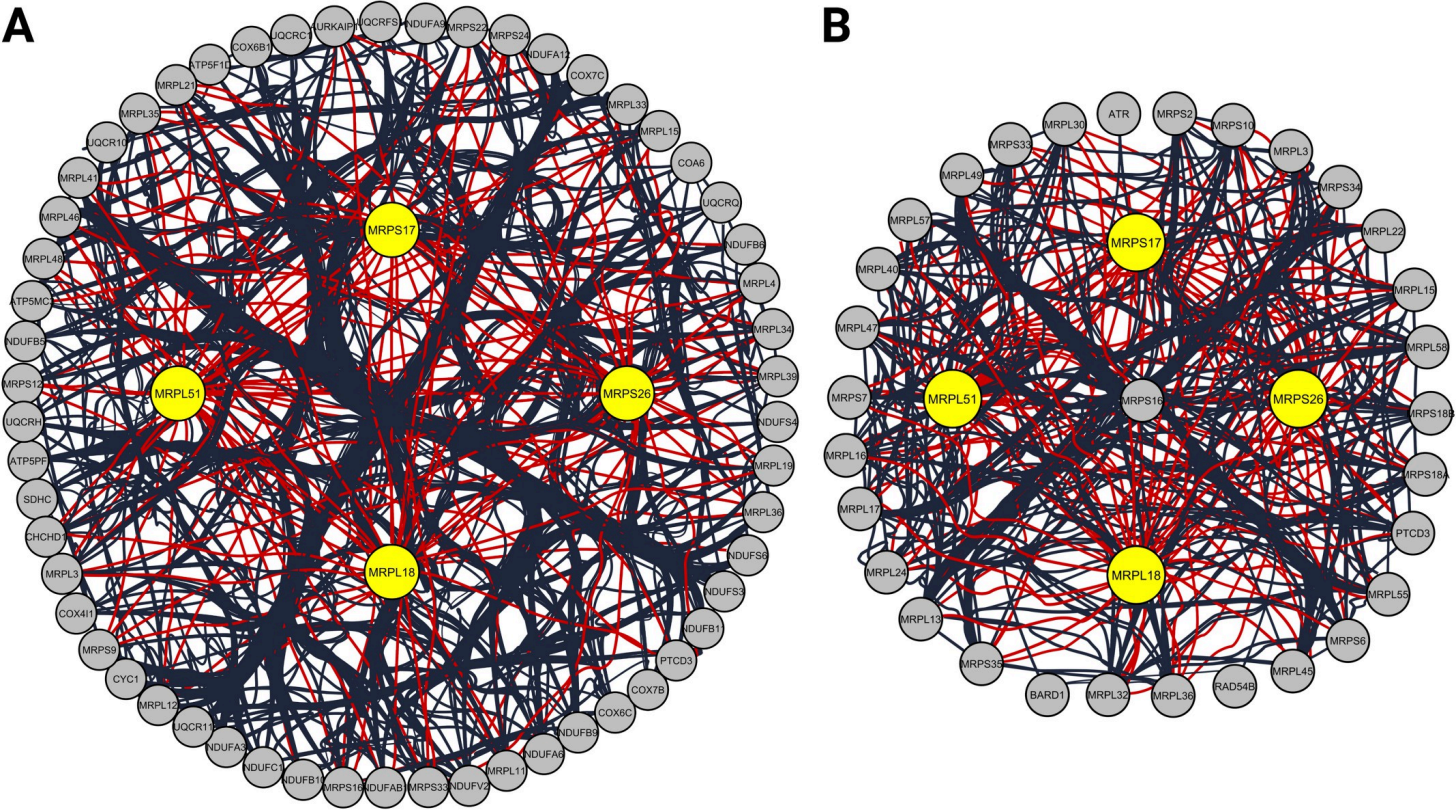
Hub genes of clustering modules in the protein-protein interaction network of differentially expressed genes from (A) musculoskeletal ageing and (B) non-small cell lung cancer (NSCLC) patients, that were linked with reduced overall survival, opposing tissue expression to musculoskeletal ageing and tumour purity in patients with NSCLC from public transcriptome data. Yellow nodes constitute hub genes. MRPL18: Mitochondrial Ribosomal Protein L18; MRPL51: Mitochondrial Ribosomal Protein L51; MRPS17: Mitochondrial Ribosomal Protein S17; MRPS26: Mitochondrial Ribosomal Protein S26.

Two PPI networks of DEGs from musculoskeletal ageing and NSCLC datasets were created and consisted of a total of 1763 and 4192 DEGs along 13436 and 66041 interactions, respectively. Two highly clustered gene modules were identified in the musculoskeletal ageing network and four in the NSCLC one (Table 1). The five highest-ranked hub module genes present across both networks, were retrieved: Mitochondrial Ribosomal Protein S26 (MRPS26), Mitochondrial Ribosomal Protein S17 (MRPS17), Mitochondrial Ribosomal Protein L18 (MRPL18), Mitochondrial Ribosomal Protein L51 (MRPL51) and Coiled-Coil-Helix-Coiled-Coil-Helix Domain Containing 1 (CHCHD1) (Tables 2 and 3, Fig 2).

**Table 1:** Gene composition of the highest-ranked clustering modules in the protein-protein interaction network of differentially expressed genes of musculoskeletal ageing and non-small cell lung cancer.

Cluster	MCODE score	Gene density	Gene edges	Genes
*Musculoskeletal ageing*				
1	32.542	60	960	ATP5F1D, ATP5MC3, ATP5PF, AURKAIP1, CHCHD1, COA6, COX4I1, COX6B1, COX6C, COX7B, COX7C, CYC1, MRPL11, MRPL12, MRPL15, MRPL18, MRPL19, MRPL21, MRPL3, MRPL33, MRPL34, MRPL35, MRPL36, MRPL39, MRPL4, MRPL41, MRPL46, MRPL48, MRPL51, MRPS12, MRPS16, MRPS17, MRPS22, MRPS24, MRPS26, MRPS33, MRPS9, NDUFA12, NDUFA3, NDUFA6, NDUFA9, NDUFAB1, NDUFB10, NDUFB11, NDUFB5, NDUFB6, NDUFB9, NDUFC1, NDUFS3, NDUFS4, NDUFS6, NDUFV2, PTCD3, SDHC, UQCR10, UQCR11, UQCRC1, UQCRFS1, UQCRH, UQCRQ
2	17.000	19	153	C18orf32, EEF1G, EIF1AX, EPRS1, MTRF1, NHP2, RPL17, RPL23A, RPL26L1, RPL30, RPL36A, RPL3L, RPLP0, RPS10, RPS18, RPS3, RPSA, RSL24D1, UBXN7
*Non-small cell lung cancer*				
1	96.523	112	5357	ANLN, ASF1B, ASPM, ATAD2, BIRC5, BUB1, CCN1, CCNB1, CCNB2, CCNE2, CDC20, CDC25C, CDC45, CDC6, CDCA2, CDCA3, CDCA5, CDCA7, CDCA8, CDK1, CDKN3, CENPA, CENPE, CENPF, CENPK, CENPM, CENPU, CEP55, CHEK1, CKAP2L, CKS1B, DEPDC1, DEPDC1B, DLGAP5, DTL, E2F7, ERCC6L, ESPL1, EXO1, EZH2, FAM83D, FANCI, FBXO5, FEN1, FOXM1, GINS1, GINS2, GMNN, GTSE1, HJURP, HMMR, KIF11, KIF14, KIF15, KIF18A, KIF18B, KIF20A, KIF23, KIF2C, KIF4A, KNL1, KNTC1, MCM10, MCM2, MCM6, MELK, MKI67, MND1, MYBL2, NCAPD2, NCAPG, NCAPG2, NCAPH, NDC80, NEIL3, NEK2, NUF2, OIP5, ORC1, PARPBP, PBK, PCLAF, PCNA, PIMREG, PLK1, POLE2, PRC1, PRIM1, PTTG1, RACGAP1, RAD51, RAD51AP1, RAD54L, RFC4, RRM1, RRM2, SHCBP1, SKA3, SMC2, SPC25, STIL, TACC3, TK1, TOP2A, TPX2, TRIP13, TROAP, TTK, UBE2C, UBE2T, WDHD1, ZWINT
2	31.380	72	1114	AATF, ABT1, BMS1, BOP1, CTPS1, DCAF13, DDX51, DDX54, DDX56, DHFR, DHX32, DKC1, DTYMK, EARS2, EBNA1BP2, EIF5A, EIF6, FANCA, FTSJ1, FTSJ3, GNL1, GNL2, GNL3, GNL3L, HEATR1, HUS1, LSG1, MDC1, MTIF2, NOB1, NOC4L, NOL6, NOP14, NVL, ORC5, OXA1L, POLD2, RAD51C, RECQL4, RIOK2, RPA3, RPF2, RPL10A, RPL12, RPL13, RPL15, RPL19, RPL22L1, RPL26L1, RPL30, RPL31, RPL39L, RPL6, RPL7, RPL8, RPP38, RPS18, RPS19, RPS24, RRP1, RRS1, SIL1, TOPBP1, TRMT1L, TRMT2B, TUFM, URB1, UTP25, UTP4, WDR12, WDR3, WDR74
3	30.400	36	532	ATR, BARD1, CHCHD1, MRPL13, MRPL15, MRPL16, MRPL17, MRPL18, MRPL22, MRPL24, MRPL3, MRPL30, MRPL32, MRPL36, MRPL40, MRPL45, MRPL47, MRPL49, MRPL51, MRPL55, MRPL57, MRPL58, MRPS10, MRPS16, MRPS17, MRPS18A, MRPS18B, MRPS2, MRPS26, MRPS33, MRPS34, MRPS35, MRPS6, MRPS7, PTCD3, RAD54B
4	17.195	88	748	ACE, AOC3, CASP1, CAV1, CCL19, CCL22, CCL4, CCR1, CCRL2, CD19, CD27, CD274, CD276, CD69, CDH1, CDH5, CDKN1A, CENPL, CENPQ, CENPX, CSF3, CTPS2, CX3CL1, CXCL1, CXCL13, CXCL16, CXCL3, CXCL6, CXCL9, CXCR2, ELP3, FANCG, FANCL, FAS, FCGR2A, FCGR3B, GUF1, H2AC6, H2AC7, H2AJ, H2BC12, H2BC5, H2BC9, HAVCR2, HGH1, HMGB1, HMOX1, IL1A, IL2RA, IL33, IL7R, IPO4, ITGAE, ITGAX, JUN, KLRC4-KLRK1, METTL1, MIS18A, MMP13, MMP7, MRM2, NFKBIA, PALB2, PARP1, PF4, PKM, PLAU, POLR2C, PRF1, PUS7, RAD1, RBBP7, RBBP8, RMI1, SELE, SELP, SOCS3, STN1, TBX21, TEK, TFB1M, TFDP1, THBS1, TIMP1, TLR4, TRUB2, TTC4, VCAM1

MCODE: Molecular Complex Detection.

**Table 2:** The top five ranked and overlapping hub genes according to 11 topological algorithms in the protein-protein interaction networks of musculoskeletal ageing and non-small cell lung cancer differentially expressed genes.

Gene ID	Musculoskeletal ageing		Non-small cell lung cancer		Gene name
	*P*-value	*Z*-score	*P*-value	*Z*-score	
MRPS26	1.05E-02	-3.46	4.79E-02	2.45	Mitochondrial Ribosomal Protein S26
MRPS17	4.09E-02	-2.92	2.73E-02	2.78	Mitochondrial Ribosomal Protein S17
MRPL18	2.78E-05	-5.18	2.15E-02	2.92	Mitochondrial Ribosomal Protein L18
MRPL51	9.26E-03	-3.51	4.95E-02	2.43	Mitochondrial Ribosomal Protein L51
CHCHD1	4.88E-02	-2.84	4.47E-02	2.49	Coiled-Coil-Helix-Coiled-Coil-Helix Domain Containing 1

CHCHD1: Coiled-Coil-Helix-Coiled-Coil-Helix Domain Containing 1; MRPL18: Mitochondrial Ribosomal Protein L18; MRPL51: Mitochondrial Ribosomal Protein L51; MRPS17: Mitochondrial Ribosomal Protein S17; MRPS26: Mitochondrial Ribosomal Protein S26.

**Table 3:** Five highest-ranked hub genes according to 11 topological algorithms ranked in the protein-protein interaction network of differentially expressed genes between musculoskeletal ageing and non-small lung cancer. Numbers represent score.

Topological Score	MRPS26	MRPS17	MRPL18	MRPL51	CHCHD1
*Musculoskeletal ageing*					
MCC	9.22E+13	9.22E+13	9.22E+13	9.22E+13	9.22E+13
DMNC	1.16	1.11	1.09	1.02	1.09
MNC	37.00	39.00	40.00	43.00	39.00
Degree	37.00	41.00	42.00	45.00	43.00
EPC	158.89	180.97	173.98	166.69	161.45
BottleNeck	1.00	1.00	1.00	1.00	2.00
EcCentricity	0.20	0.20	0.20	0.20	0.20
Closeness	1408.72	1444.37	1435.73	1463.67	1413.98
Radiality	5.88	5.95	5.93	5.99	5.88
Betweenness	723.45	2364.60	2161.21	3977.35	3270.41
Stress	21072.00	51878.00	47274.00	88412.00	64944.00
*Non-small cell lung cancer*					
MCC	9.22E+13	9.22E+13	9.22E+13	9.22E+13	9.22E+13
DMNC	1.02	1.06	1.13	1.17	1.00
MNC	33.00	32.00	31.00	32.00	33.00
Degree	33.00	32.00	32.00	35.00	35.00
EPC	40.53	40.14	40.30	39.91	37.89
BottleNeck	1.00	1.00	2.00	1.00	2.00
EcCentricity	0.17	0.17	0.17	0.17	0.17
Closeness	588.33	574.20	575.63	600.20	577.40
Radiality	7.76	7.67	7.68	7.80	7.68
Betweenness	1224.50	340.27	676.94	1904.13	769.72
Stress	23148.00	7994.00	12340.00	31242.00	16996.00

CHCHD1: Coiled-Coil-Helix-Coiled-Coil-Helix Domain Containing 1; DMNC: Density of Maximum Neighborhood Component; EPC: Percolated Component; MCC: Maximal Clique Centrality; MNC: Maximum Neighborhood Component; MRPL18: Mitochondrial Ribosomal Protein L18; MRPL51: Mitochondrial Ribosomal Protein L51; MRPS17: Mitochondrial Ribosomal Protein S17; MRPS26: Mitochondrial Ribosomal Protein S26.

### Prognosis, expression level and tumour purity of musculoskeletal ageing hub genes in NSCLC

**Fig 3:**
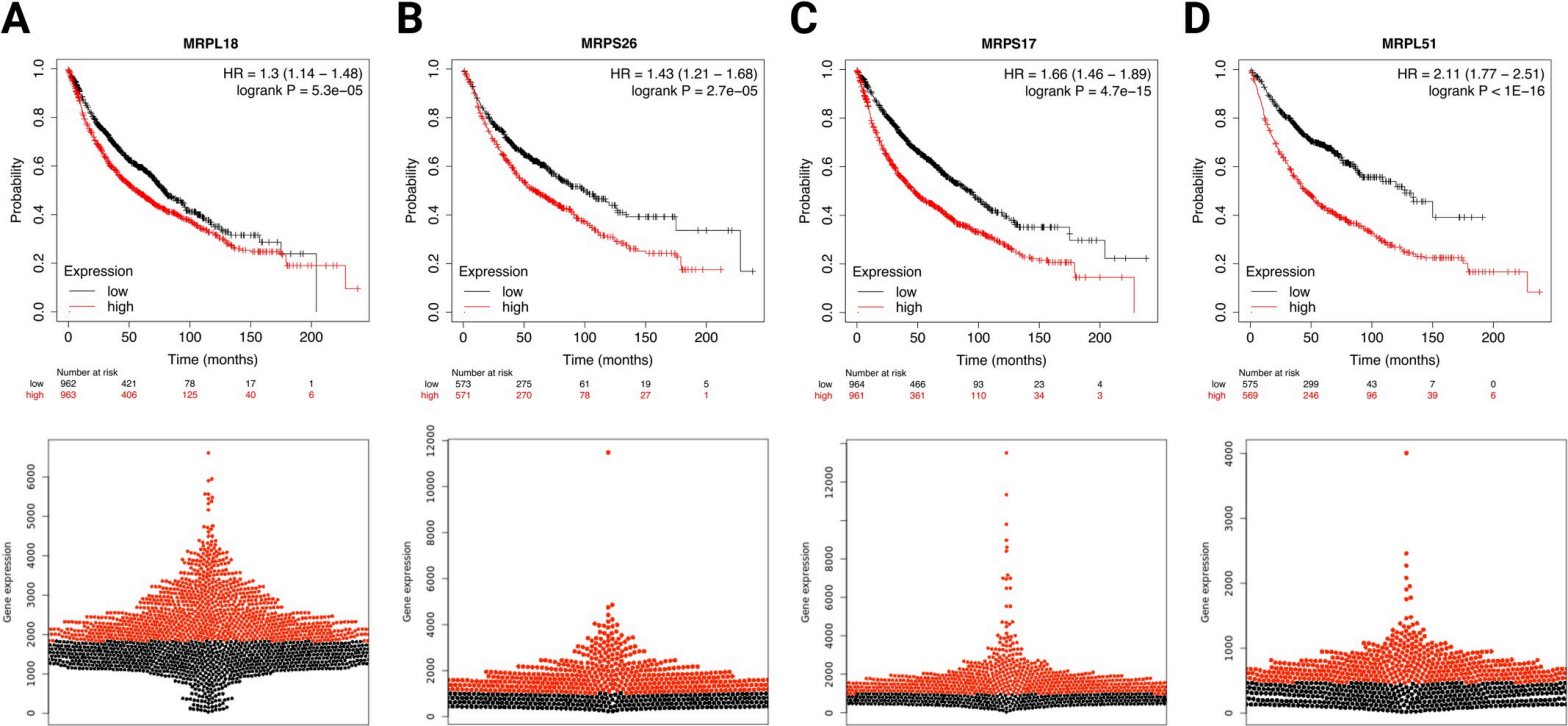
Association of Mitochondrial Ribosomal Protein S26 (MRPS26), Mitochondrial Ribosomal Protein S17 (MRPS17), Mitochondrial Ribosomal Protein L18 (MRPL18) and Mitochondrial Ribosomal Protein L51 (MRPL51) expression with overall survival in non-small cell lung cancer patients. Significance was determined using a log-rank *P*<0.05 and the corresponding beeswarm graphs of probe distribution were displayed. HR: Hazard ratio.

**Fig 4:**
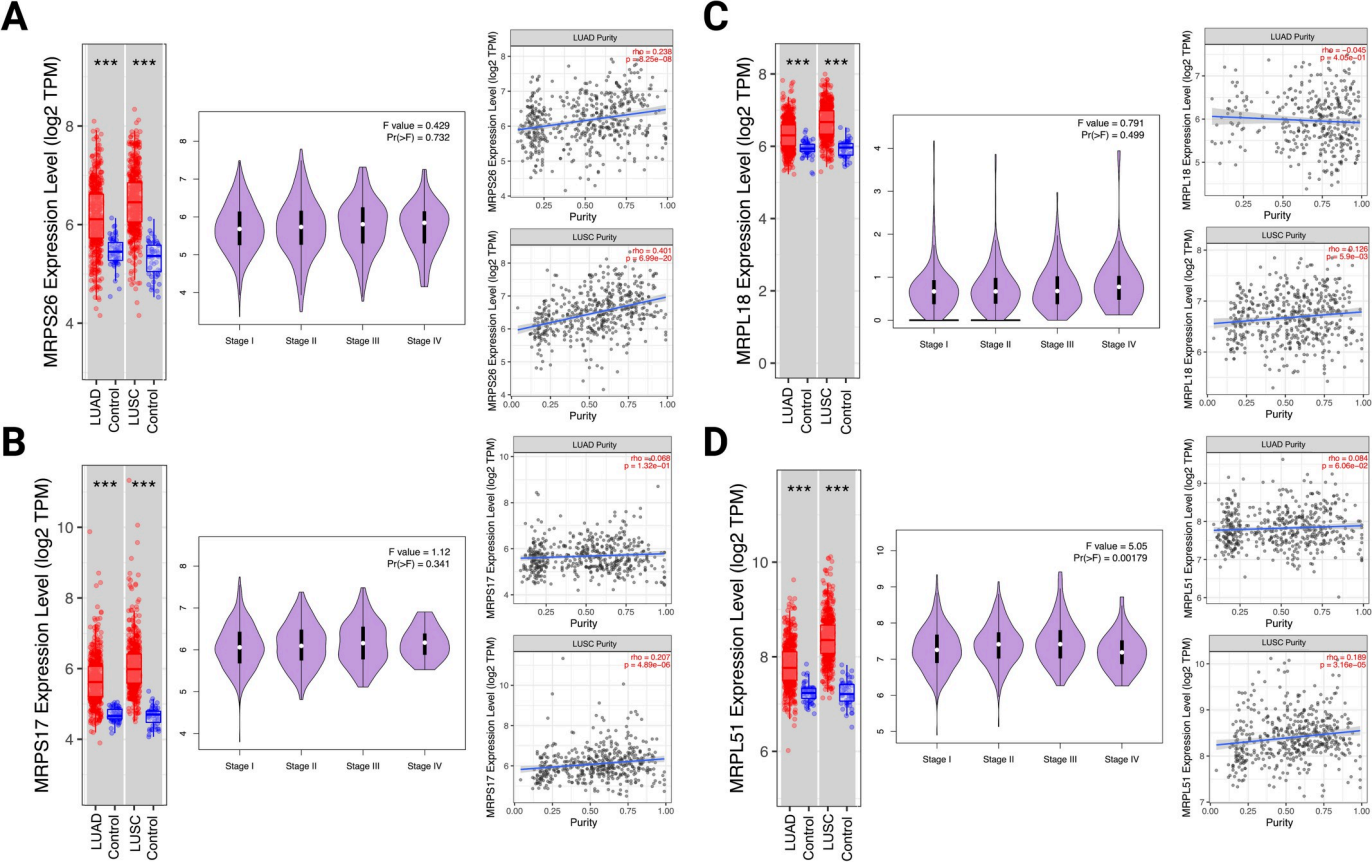
Overall expression, across different stages and in terms of tumour purity of Mitochondrial Ribosomal Protein S26 (MRPS26), Mitochondrial Ribosomal Protein S17 (MRPS17), Mitochondrial Ribosomal Protein L18 (MRPL18) and Mitochondrial Ribosomal Protein L51 (MRPL51) in non-small cell lung cancer tissues from public transcriptome data. Significance was determined using analysis of variance, a Wilcoxon test P<0.05 and the partial Spearman’s correlation (rho).*** *P*<0.001. LUAD: Lung adenocarcinoma; LUSC: Lung squamous cell carcinoma; TPM: Transcripts per million.

Survival analysis of the GEO, TCGA and caArray revealed that high (but opposite to musculoskeletal ageing) expression of MRPS26, MRPS17, MRPL18 and MRPL51 correlated with significantly reduced OS (MRPS26: log-rank *P* = 2.7E-05, HR = 1.43; MRPL18: log-rank *P* = 5.3E-05, HR = 1.3; MRPS17: log-rank *P* = 4.7E-15, HR = 1.66; MRPL51: log-rank *P*<1E-16, HR = 2.11) in patients with NSCLC (Fig 3). Overall expression of these genes was significantly upregulated in NSCLC tissues when compared to control, but with MRPL18 having also altered expression between earlier and advanced disease states. The expression levels of these genes (apart from MRPS17, MRPL18 MRPL51 in LUAD) were correlated with tumour purity in NSCLC (LUAD: MRPS26: *P* = 8.25E-08, partial rho = 0.238; LUSC: MRPS26: *P* = 6.99E-20, partial rho = 0.401; MRPS17: *P* = 4.89E-06, partial rho = 0.207; MRPL18: *P* = 5.9E-03, partial rho = 0.126; MRPL51: *P* = 3.16E-05, partial rho = 0.189) (Fig 4).

## Discussion

**Fig 5:**
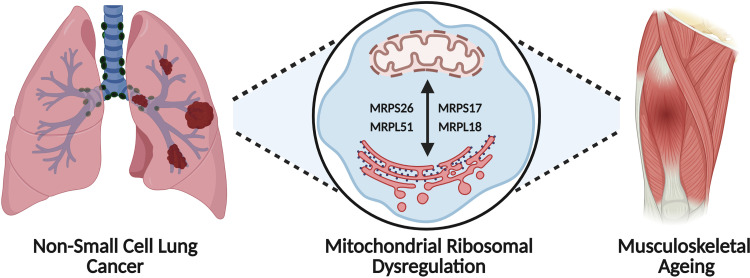
Dysregulated expression of mitochondrial ribosomal protein genes, Mitochondrial Ribosomal Protein S26 (MRPS26), Mitochondrial Ribosomal Protein S17 (MRPS17), Mitochondrial Ribosomal Protein L18 (MRPL18) and Mitochondrial Ribosomal Protein L51 (MRPL51), as marker of musculoskeletal ageing and non-small cell lung cancer disease progression.

This study aimed to identify gene markers whose dysregulated expression and protein interaction interference may potentially be involved in both musculoskeletal ageing and NSCLC cachexia. Analysis of differentially expressed genes of musculoskeletal samples from healthy older adults and lung tissues from patients with NSCLC, identified two gene modules in the musculoskeletal ageing network and four in the NSCLC one. Multi-algorithmic topological analysis revealed four overlapping mammalian MRP hub genes, MRPS26, MRPS17, MRPL18, and MRPL51, with opposing tissue expression between musculoskeletal ageing and NSCLC when tissues from both groups were compared to their corresponding control states [i.e., a) older healthy vs younger healthy and b) NSCLC vs age-matched healthy]. The dysregulated expression of these genes was specifically linked with reduced OS and tumour purity in patients with NSCLC. Our findings highlight the potential significant role of mitochondrial ribosomal genes as potential markers of musculoskeletal ageing and NSCLC disease progression (Fig 5).

NSCLC is associated with abnormal expression of multiple MRP genes, and the downregulation of key transcriptional factors involved in mitochondrial biogenesis have been described during muscle atrophy [57, 58]. Furthermore, mitochondrial dysfunction has been suggested to play a key role for the onset and progression of sarcopenia [18], while mitochondrial dysfunction either due to impaired mitochondrial protein synthesis or mitoribosome misassembly can initiate mitochondrial ribosomal stress and be a contributing factor to diseases such as lung cancer [59]. For instance, MRPS26 has been linked with intrinsic apoptotic pathway signalling and DNA damage response as a result of perturbations in p53 [60]. Indeed, the involvement of MRPS26 has been observed in mitochondrial activity of muscle stem cells [61], hinting that its expression could be central in mitochondrial degeneration during muscle atrophy-induced cancer cachexia [58].

MRPS17 is another gene that is consistently expressed in multiple cancers, including NSCLC [62, 63]. The upregulation of MRPS17 has been linked with resistance to chemotherapy treatment as described in trials with anti-cancer agents, temozolomide and nitrosoureas [64]. Furthermore, a more recent human study has revealed that MRPS17 promotes gastric cancer metastasis through abnormal signaling of phosphatidylinositol-3 kinase/Akt (PI3K/Akt) [64], a pathway commonly dysregulated in NSCLC [65]. In muscle bioenergetics, PI3K/Akt dysregulation may induce muscle atrophy by enhancing myostatin, and suppress muscle hypertrophy via inhibiting the phosphorylation of mammalian target of rapamycin complex 1 signaling [66]. Future research is warranted to confirm the role of musculoskeletal MRPS17 in PI3K/Akt metabolism and its contribution in ageing humans with NSCLC.

Dysregulated expression of MRPL18 has been correlated with tumour progression [67], and its co-occurrence with NSCLC via Heat-Shock Factor 1 activation [68] has been previously described. Prominently, perturbations in MRPL18 have been linked with cytosolic stress response in insulin resistant microenvironments [69]. Additionally, abnormal MRPL18 responses have been involved with peroxisome proliferator-activated receptor γ coactivator-1β deficient mice in which dissociation between mitochondrial dysfunction and insulin resistance has been revealed [70]. Thus, musculoskeletal MRPL18 may be central in facilitating muscle oxidation, [70] hinting a possible association between impaired muscle oxidative capacity, physical performance, and insulin resistance in ageing and cachexia-related NSCLC [71].

Dysfunctional mitochondrial translation has also been linked with MRPL51 dysregulation [72, 73]. Although human evidence in the setting of tumour progression remains unexplored, there are emerging findings of its upregulation in NSCLC cell models, whereby Maiuthed *et al*. [74] demonstrated >5-fold increase in MRPL51 in a lung cancer cell model. Attributed to its elemental role in mitochondrial bioenergetics, the ramification of MRPL51 with myofibre growth [75], muscle disuse atrophy [76], myostatin regulation [77], and dysregulated oxidative phosphorylation [78] has been described. Therefore, it may be speculated that mitochondrial alterations in adenosine triphosphate (ATP) synthesis and mitochondrial uncoupling in skeletal muscle may be associated muscle wasting in ageing, that could possibly instigate cancer-related cachexia.

Taken all of the above into consideration, mitochondrial dysfunction via the dysregulated expression of mammalian MRPs, possibly leads to insufficient ATP production during muscle regeneration and may underline a link between muscle wasting during ageing and cachexia-related cancer [79]. However, the interplay between mitochondrial dysfunction in skeletal muscle among older adults may be also mediated by the accumulation of visceral fat and insulin resistance [80]. Glucose metabolism is largely dependent on mitochondrial activity for cellular energy production, thereby fat accumulation in skeletal muscle may reduce mitochondrial oxidative and phosphorylation capacity [81]. Specifically, the accretion of reactive oxygen species by an overwhelming inflation of nicotinamide adenine dinucleotide phosphate oxidases via adipocytes and/or macrophages, may contribute to inflammation in the adipose tissue [82]. Elevated levels of low-grade inflammation are accompanied by a concomitant decrease of myoglobin and increased atrogenes (i.e., MuRF1, Atrogin-1) in lung cancer models, that are involved in skeletal muscle degradation [83]. In this context, research in late-onset obesity rats has reported truncation of mitochondrial assembly factors being associated with increased adiposity and dysregulated insulin signaling [84]. Nevertheless, experimental human studies investigating the relationship of MRPs and their expression in musculoskeletal ageing and NSCLC are required to explore the underlying genetic links between sarcopenia and cachexia-related cancer. Importantly, the role of skeletal muscle satellite cells as regulators of hypertrophic [85–88] and non-hypertrophic [89, 90] tissue remodeling should not be dismissed since satellite cells in older sarcopenic adults influences the ability of skeletal muscle to regenerate, repair, and remodel [91], while a decline in the overall number of satellite cells may be more prevalent in aged muscle [92, 93]. Reduced satellite cell content has been particularly observed in type II muscle fibres of older adults [94–98]. Considering that in cancer, glycolysis becomes more important for energy provision due to a reduced mitochondrial OXPHOS capacity [26], future studies should also further explore the links between dysregulated expression of glycolytic genes in NSCLC and musculoskeletal ageing. Regarding satellite cell function, Fausnacht *et al*. [99] observed that diet had a greater influence on satellite cell function than ageing, however their study was *in vitro* and ageing still influenced (alongside body mass index) the response of satellite cells depending on substrate availability.

This is the first study that examined the potential role of DEGs and their interactome as gene biomarkers in musculoskeletal ageing and NSCLC, using 14 publicly available datasets with a total of 1156 participants. By doing so, we applied a multi-algorithmic protein-interaction based strategy which employed diverse levels of filtering beyond gene expression.

Our study was also prone to limitations. Although expression profiling by array of the included datasets was performed using similar platforms, studies with heterogeneous platform use were excluded, preventing the more powerful and reliable detection of potential DEGs. Even then, lab effects have a known impact in gene profiling which often results in different scales of measurement that inevitably lowers the number of integrated DEGs [100]. Evident of this is the difference in DEGs between the musculoskeletal ageing and NSCLC datasets, with the former group being less than 45% in total DEG count. However, this is a well-described drawback in the literature and experimental variation between labs may prevail, even following normalization [101–104]. Lastly, it was not feasible to control for other potential confounders in gene expression such as demographic characteristics (e.g. sex, age, race), clinicopathological characteristics beyond cancer stage (e.g. nodal metastasis status) and medical comorbidities (e.g., obesity) in the patients with NSCLC from the included datasets, which hinders the true association with musculoskeletal ageing [105, 106].

The physiological burden of age-related muscle dysfunction due to increased prevalence of sarcopenia and NSCLC remains a challenge. Studies identifying key genes that mediate the sarcopenia-cancer cachexia crosstalk may provide valuable insight in developing targeted pharmacological and/or exercise and nutritional interventions, aiming to promote higher quality of life and alleviate poor outcomes. Our study showed that MRPs, MRPS26, MRPS17, MRPL18, and MRPL51 exhibited multi-algorithmic topological significance among DEGs from musculoskeletal ageing and NSCLC samples, suggesting the potential involvement of the mitochondrial microenvironment as a link between these conditions. Experimental studies in humans are warranted to validate the diagnostic and prognostic value of MRPs in older patients with sarcopenia and NSCLC progression.

## Supporting information

S1 Table Characteristics of the gene expression datasets included in the analysis. (DOCX)

S2 Table Differentially expressed genes of musculoskeletal samples between older (≥ 60 years of age) and young (≤ 30 years of age) adults. (XLSX)

S3 Table Differentially expressed genes of lung samples between patients with non-small cell lung cancer and healthy age-matched controls or matched adjacent / distant normal lung tissue form the same patient. (XLSX)

S4 Table Overlapping differentially expressed genes of muscle and lung samples between musculoskeletal ageing and non-small cell lung cancer. (XLSX)
