# Supplementary material for: Aberrant mitochondrial homeostasis at the crossroad of musculoskeletal ageing and non-small cell lung cancer
Source: PLoS One. 2022 Sep 6;17(9):e0273766. doi: 10.1371/journal.pone.0273766 (PMC9447904; doi:10.1371/journal.pone.0273766)
Supplement: S1 Table — (DOCX) [file pone.0273766.s001.docx]

| **Gene expression datasets** | **Controls** | | | **Experiments** | | | **Tissue sample** | **Microarray**  **platform** |
| --- | --- | --- | --- | --- | --- | --- | --- | --- |
|  | ***n*** | **age** | **sex** | ***n*** | **age** | **sex** |  |  |
| *Musculoskeletal ageing* | | | | | | | | |
| GSE25941 | 15 | 24  -  26 | M/F | 21 | 77  -  79 | M/F | Vastus lateralis | GPL570 |
| GSE28392 | 20 | 21  -  25 | F | 14 | 84  -  86 | F | Vastus lateralis | GPL570 |
| GSE28422 | 30 | 23  -  25 | M/F | 24 | 83  -  85 | M/F | Vastus lateralis | GPL570 |
| GSE47881 | 10 | 18  -  30 | M/F | 16 | 60  -  75 | M/F | Vastus lateralis | GPL570 |
| GSE47969 | 6 | 24  -  30 | M/F | 22 | 60  -  68 | M/F | Vastus lateralis | GPL570 |
| GSE59880 | 15 | 19  -  28 | M | 13 | 59  -  77 | M | Vastus lateralis | GPL570 |
| *Non-small cell lung cancer* | | | | | | | | |
| GSE118370 | 6 | <60  -  ≥60 | M/F | 6 | <60  -  ≥60 | M/F | Adenocarcinoma (6)  Tumour stage I (4), II (2)  Matched adjacent normal tissue | GPL570 |
| GSE33532 | 20 | 38  -  69 | M/F | 80 | 38  -  69 | M/F | Adenocarcinoma (11)  Squamous-cell carcinomas (4)  Mixed-type carcinoma (5)  Stage IA (4), IB (10), IIA (1), IIB (5)  Matched distant normal tissue | GPL570 |
| GSE19804 | 60 | 51  -  71 | F | 60 | 51  -  71 | F | Adenocarcinoma (56)  Bronchioloaveolar carcinoma (3)  Squamous carcinoma (1)  Tumour stage I/II (47) III/IV (13)  Matched adjacent normal tissue | GPL570 |
| GSE18842 | 45^*^ | NR | NR | 46 | NR | NR | Adenocarcinoma (14)  Squamous-cell carcinomas (32)  Tumour stage I (38), II (4), II (3), IV (1)  Matched distant normal tissue | GPL570 |
| GSE27262 | 25 | 34  -  77 | NR | 25 | 34  -  77 | NR | Adenocarcinoma (25)  Tumour stage I (25)  Matched adjacent normal tissue | GPL570 |
| GSE19188 | 65 | 51  -  71 | M/F | 91^**^ | 51  -  71 | M/F | Adenocarcinoma (32/24)  Squamous-cell carcinoma (27/16)  Large-cell carcinoma (13/24)  Bronchioloalveolar / Carcinoid / Mixed adeno-squamous, or other (19/27)  Tumour stage I (51), II (21), III/IV (10)  Matched adjacent normal tissue | GPL570 |
| GSE31210 | 20^***^ | 30  -  89 | M/F | 226 | 30  -  76 | M/F | Adenocarcinoma (226)  Tumour stage I (168), II (58) | GPL570 |
| GSE40791 | 100 | 31  -  79 | M/F | 94 | 41  -  93 | M/F | Adenocarcinoma (94)  Tumour stage I (69), II (12), III (13) | GPL570 |

NR: Non-reported; ^*^Five samples were corresponding nontumor samples; ^**^Tumour tissues were assessed by two reviewers; ^***^Fifteen samples were corresponding nontumor samples.
